# Ensemble model for rail surface defects detection

**DOI:** 10.1371/journal.pone.0268518

**Published:** 2022-05-17

**Authors:** Hailang Li, Fan Wang, Junbo Liu, Haoran Song, Zhixiong Hou, Peng Dai

**Affiliations:** Railway Infrastructure Inspection Institute, China Academy of Railway Science, Beijing, China; University of Birmingham, UNITED KINGDOM

## Abstract

The detection of rail surface defects is vital for high-speed rail maintenance and management. The CNN-based computer vision approach has been proved to be a strong detection tool widely used in various industrial scenarios. However, the CNN-based detection models are diverse from each other in performance, and most of them require sufficient training samples to achieve high detection performance. Selecting an appropriate model and tuning it with insufficient annotated rail defect images is time-consuming and tedious. To overcome this challenge, motivated by ensemble learning that uses multiple learning algorithms to obtain better predictive performance, we develop an ensemble framework for industrialized rail defect detection. We apply multiple backbone networks individually to obtain features, and mix them in a binary format to obtain better and more diverse sub-networks. Image augmentation and feature augmentation operations are randomly applied to further make the model more diverse. A shared feature pyramid network is adopted to reduce model parameters as well as computation cost. Experimental results substantiate that the approach outperforms single detecting architecture in our specified rail defect task. On the collected dataset with 8 defect classes, our algorithm achieves 7.4% higher mAP.5 compared with YOLOv5 and 2.8% higher mAP.5 compared with Faster R-CNN.

## Introduction

In the high-speed railway system, the rail plays a dual role of carrying and guiding the running of the train. Its performance directly affects the safety of railway transportation. Therefore, the steel rail is required to be clean and free of surface defects. However, surface defects are inevitable, initiated by degradation, temperature differences, fatigue loading, and foreign objects between the wheel and rail during train operation [1, 2], then propagated through repeated extrusion caused by the contact stress between the wheel and the rail [3]. If not detected at an early stage, rail surface defects can result in rapid deterioration and possible failure incurring high maintenance costs [4].

Early rail surface defect detection relies on manual inspection, which is inefficient and inadequate to meet the advanced high-speed railway industry [5]. Later detection methods include nondestructive evaluation (eddy current, ultrasonic wave, or acoustic emission) [6], time-frequency analysis [4], vision-based approaches [7–9], and the combination of the above [10]. Those aforementioned methods have limited effectiveness for rail surface defect detection due to the lack of ample heuristic structure information or texture features [11]. The rise and development of machine learning provides a new effective approach for rail defect detection. DNNs have been successfully adopted to detect rail corrugation [12], rail flat [13], and have been applied to investigate the condition of railway sleepers [14, 15], settlement/dipped joints [16], and other rail track components [17]. Recently, object detection has achieved a substantial breakthrough by using Convolutional Neural Networks (CNNs), and has been introduced for rail surface defect detection in the past decades [11, 18, 19]. Compared to DNNs that require various information (vibration data, frequency data, etc), vision-based approach requires only image data and is more intuitive in rail surface defect detection tasks. Typically, CNN-based approaches are usually developed based on images consistently taken by cameras mounted on rail inspection vehicles. These images are annotated manually to train CNN models in an end-to-end manner. Once fully trained, CNN models are then deployed to hardware platforms on the train and can automatically detect surface defects.

Specifically, two main difficulties are preventing CNN-based methods from being applied in the field. First, the detection accuracy is highly sensitive to the image quality, which is largely affected by the illumination and running speed of the rail inspection vehicles. Early optical cameras can take images of good quality only with low running speed during the day [20]. Later laser line scan cameras have overcome this shortcut, they can take photographs under various conditions and can suppress specular reflections [21, 22]. More accurate detection results can be achieved by introducing extra information, e.g., 3-D information [23]. Second, CNN models usually require a sufficient number of training samples to be fully trained. The widely used approach for most industrial detection applications is to follow the pretrain-finetuning paradigm [18], i.e., to completely or partially pretrain a model on large-scale public datasets such as ImageNet [24] or MS COCO [25] and then fine-tune it on rail surface defect datasets to achieve task-specific detection ability. However, annotated data are either tedious or costly in rail surface defect detection applications. The latest Rail-5k dataset for rail surface defect detection consists of only 1.1 thousand labeled defect images [26]. As a comparison, MS COCO consists of more than 16 thousand labeled images for detection. The model can hardly get enough rail surface samples for fine-tuning, thus resulting in over-fitting or a decrease in detection accuracy. Few-shot detection has shown excellent performance in various conditions [27–29] where training samples are extremely insufficient, namely one or a few images per class. However, it focuses more on improving the detection accuracy for various novel classes instead of specific defects of interest, which is less suitable for the rail defect detection scenario.

Instead of using a single model, researchers have also tried to ensemble multiple models to achieve good performance [30], especially when the training sample is insufficient [31, 32]. Previous researchers [33, 34] have empirically shown that ensembles perform better when the diversity among the models is larger. Many ensemble methods [35, 36], therefore, seek to promote diversity among their combined models. More recent studies have shown the effectiveness of ensemble methods in both classification [37, 38] and object detection [39, 40] problems. However, the process to ensemble object detectors is costly in time and memory both at training and inference, which limits its applicability.

In this paper, we develop a new rail surface defect dataset based on laser line scan cameras at high speed, and propose a novel Multi-Backbone Double Augmentation (MBDA) framework to tackle the above disadvantage. We ensemble more than one independent backbones as sub-networks within a single base model. We do not directly ensemble multiple individual sub-networks, but to construct a shared Feature Pyramid Network (FPN) [41] followed by shared detection heads after the diverse backbone feature extractors. We do this because modern detection models are usually over-parameterized [42] to achieve high enough performance. Therefore, sharing the FPN can reduce the number of the parameters of the entire model, while hardly affecting the detection performance. On the other hand, we develop two augmentation modules for input images and their extracted features respectively. Image augmentation methods are randomly selected from a developed Image Augmentation Bag (IAB), whereas feature augmentation methods are randomly selected from a developed Feature Augmentation Bag (FAB). The two augmentation operations can increase the diversity of the sub-networks, thus preventing homogenization. Finally, we test our model in a typical industrial application scenario, i.e., the rail defect detection scenario.

In summary, our contributions are two-fold:

- We propose a general framework, MBDA, connecting two successful fields: image/feature augmentations and multi-backbone ensembling. We connect sub-networks with a shared FPN to best tackle the diversity/computation cost trade-off in training and inference.
- We develop a novel feature augmentation bag to increase the diversity of sub-networks. Besides well-developed image augmentation approaches, the feature augmentation process further allows our model to perform better on extremely insufficient training data.

## Rail surface defect dataset

The rail surface defect dataset used in this paper is collected from the 9 km railway test loop built by the National Academy of Railway Sciences Test Center by a linear array camera installed on a high-speed train. Although the total number of the captured images within the dataset is more than ten thousand, only 400 of them have defect features.

Some studies categorize rail surface defects as squats, spalling, and cracks [9, 43], while others focus on wear, breakage, scour, undulation, and oxidation [8, 44]. After analyzing the collected rail surface images as well as combining the existing research and definitions, we mainly consider the following categories:

- defects
  - spalling, displacement of parent metal from the railhead.
  - scratch, small/mild wear of the lateral planes of the railhead.
  - crush, i.e., big/severe wear of the lateral planes of the railhead.
  - squat, defect initiated from rolling contact fatigue cracks.
  - crack, tear of the lateral planes of the railhead.
- dirt, paint, or mud that covers the surface of the rail.
- gap, gaps left between successive rails on a railway track.
- unknown, unrecognized features.

The unknown category includes features that cannot be recognized as any defects mentioned above, nor can they be recognized as dirt or gap. Since the unknown category usually needs extra manual recheck, we can regard it as a kind of special defect. As a result, the collected rail surface defect dataset can be used to perform an 8-category detection task. We can also perform a 3-category detection task involving the generalized defect, dirt, and gap when we concern more on whether there exists a defect or not.

**Fig 1:**
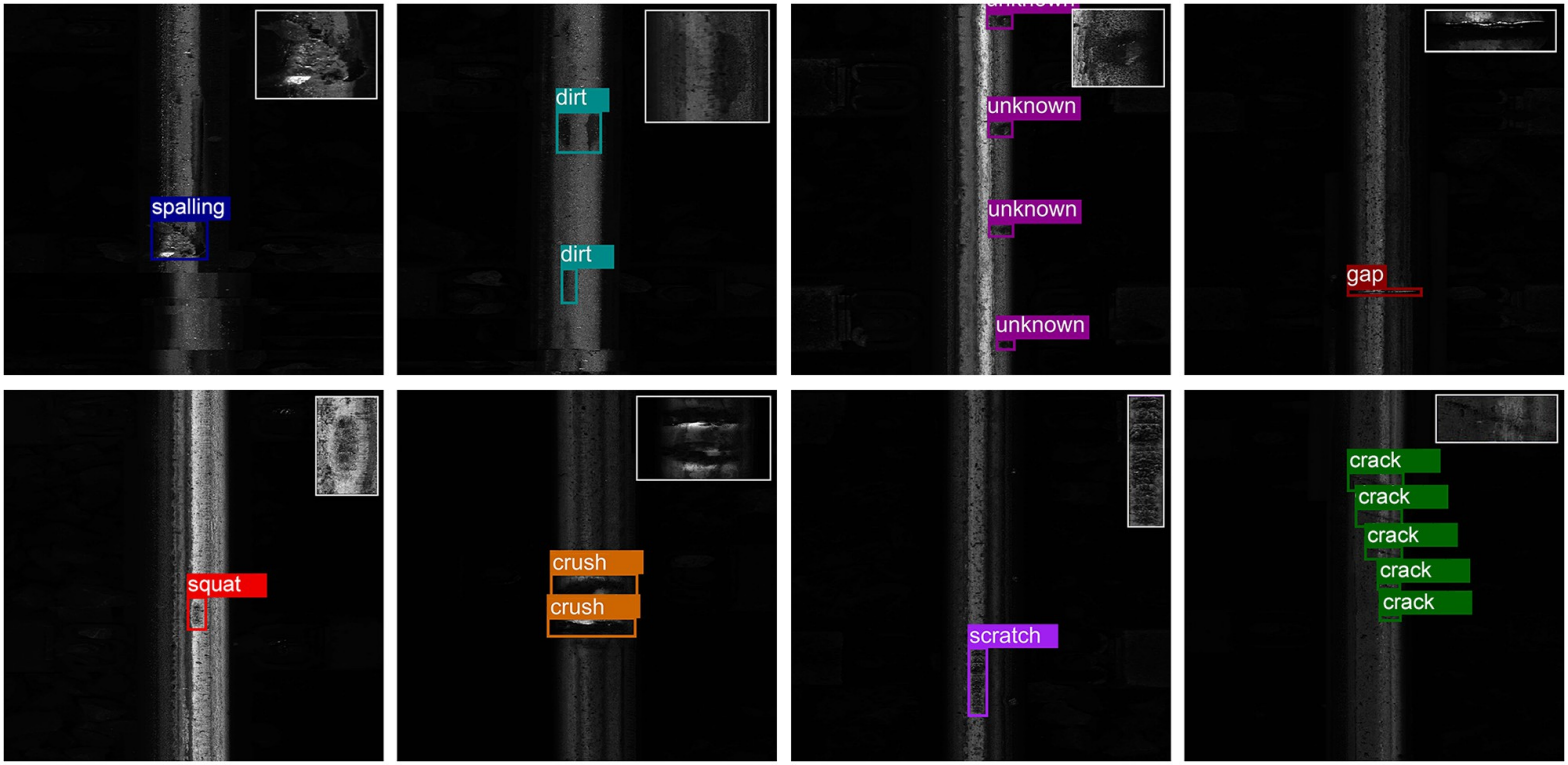
Samples of the rail defect dataset with 8 categories. From left to right, the first row contains saplling, dirt, unknown, and gap, and the second row contains squat, crush, scratch, and crack.

Examples of images in the dataset are shown in Fig 1, and enlarged examples of each category are shown in the upright corner of each subfigure. It is worth noting that the small number of annotated images makes it hard to train a detection model without over-fitting to achieve high detection performance.

## MBDA framework

**Fig 2:**
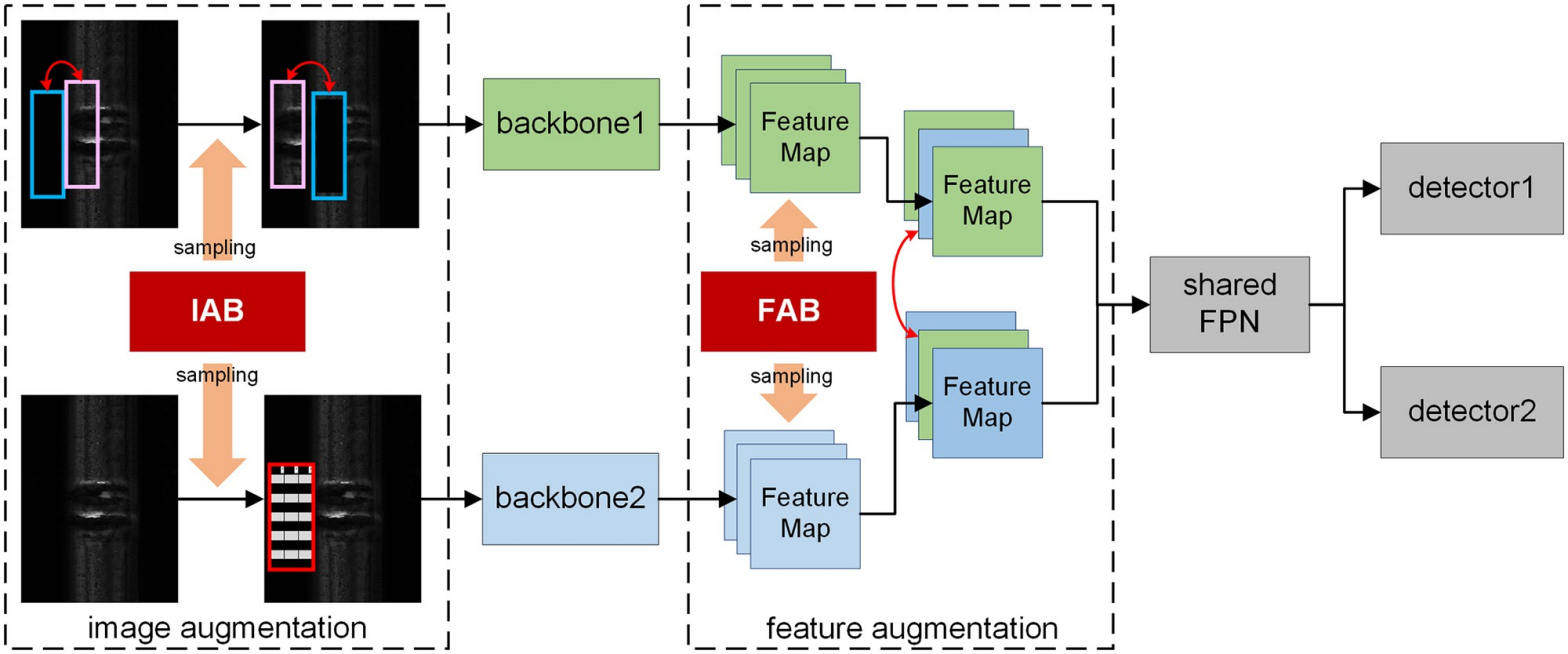
Structure of the proposed MBDA framework. The input image is a detection sample of YOLO v5.

We first introduce the overall structure of the proposed MBDA, summarized in Fig 2. Then each component of the MBDA is introduced in detail.

### Model architecture

Our MBDA roughly consists of four components as follows:

- **Image augmentation**. The image augmentation part augments the input image to obtain *N* different images by *N* data augmentation methods randomly selected from the Image Augmentation Bag (IAB).
- **Multi-backbone**. The multi-backbone component has *N* individual backbones like ResNet [45] or MobileNet [46]. The backbones used in this component can either be different from each other or share the same structure. The main diversity of the backbones lies in the random selected image and feature augmentation methods.
- **Feature augmentation**. The feature augmentation part augments features extracted by the multi-backbone component. Similar to image augmentation, feature augmentation methods are randomly selected from the Feature Augmentation Bag (FAB).
- **Shared FPN**. FPN is used to improve efficiency by concatenating the pyramid of down-sampled convolution features, and it has become a standard component in modern object detection models. We construct the shared FPN to reduce parameters and computation resource consumption.
- **Detector**. The detector component consists of *N* individual detectors for object classification and bounding box regression. Each detector is independently responsible for each corresponding backbone, which means that, in the training phase, we want each detector to make different but accurate predictions as much as possible.

During training, MBDA takes *N* augmented images as input. These images are all derived from the same training image but with different data augmentation operations. The *N* detectors are independently responsible for the detection tasks of the corresponding input images. During inference of MBDA, as a comparison, *N* identical images to be detected are taken as inputs, and an average of the *N* outputs is computed to be the category prediction result, then the Weighted Boxes Fusion [47] is used to compute the final bounding box prediction.

### Image augmentation

**Fig 3:**
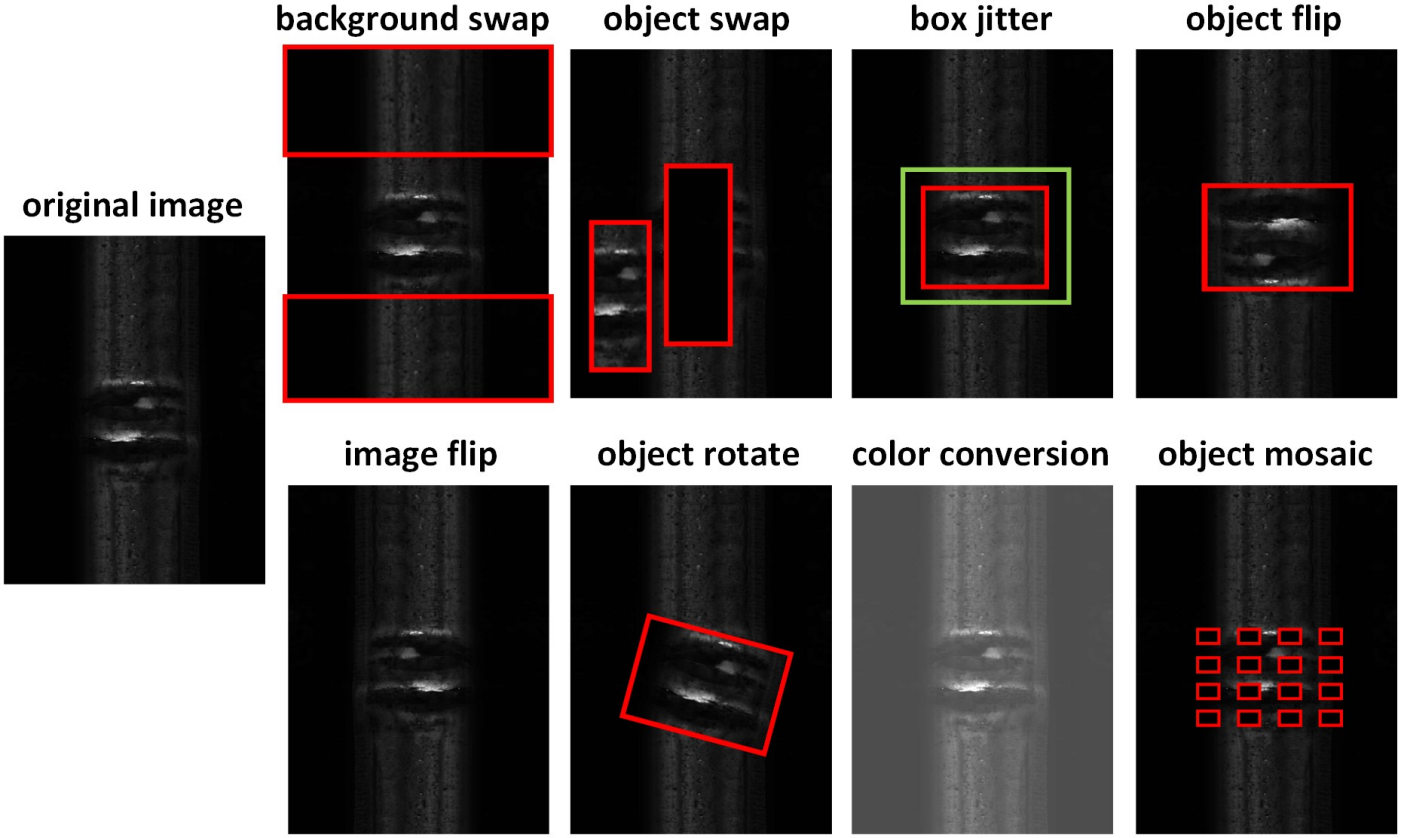
Image augmentation methods within IAB.

Data Augmentation has become a very important means to improve the performance of CNNs [48]. To improve the diversity of each sub-network, we firstly construct an Image Augmentation Bag (IAB) composed of various image augmentation methods. Then we copy each input image into *N* identical images. For each copied image, we select one image augmentation method from the IAB by sampling from a specific distribution. The sampled image augmentation method is then applied to the corresponding image, and its label is changed accordingly. Detailed image augmentation methods within the IAB are shown in Fig 3.

We use the 8 methods mentioned in Fig 3 to build the IAB. Mosaic, box dithering, and image flipping have been widely adopted as useful data preprocessing operations. Color gamut transformation, target flipping, and target rotation have also been proved to be effective in target detection [49], In order to adapt to the network structure characteristics of our MBDA, we also designed two extra augmentation operations: target swap and background swap, which make the input data of each sub-network more different, so that each detection head can extract the characteristics of the corresponding backbone and improve the diversity of the network.

### Feature augmentation

**Fig 4:**
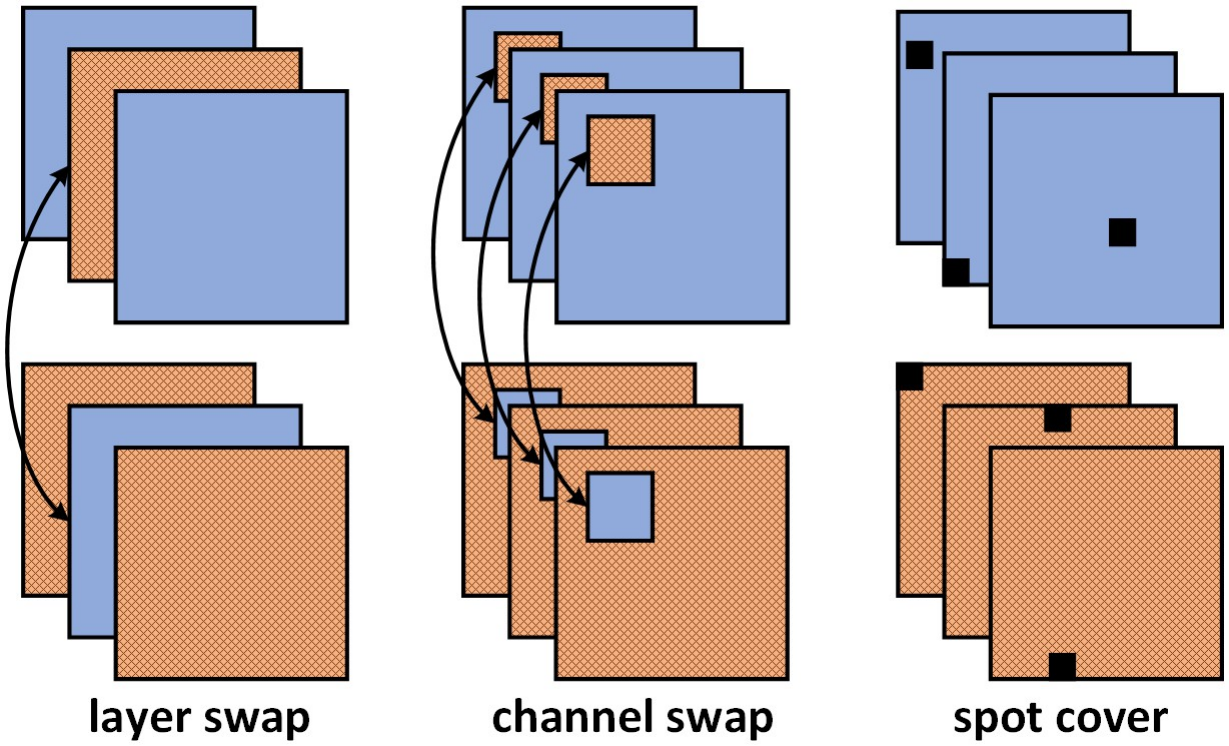
Feature augmentation methods within FAB.

Similar to image augmentation, we also construct a Feature Augmentation Bag (FAB) for feature maps extracted by the backbones. The development of FAB is inspired by MIXMO [38], which improves the diversity of sub-network by using CutMix [50], an effective Mixed Sample Data Augmentation (MSDA) method, on the feature map to improve the accuracy of image classification tasks. But unlike MIXMO, we apply feature augmentation in the object detection task, and we randomly select methods from multiple augmentation methods in the FAB instead of just one single method, which brings more diversity and thus makes the detection model perform better [51]. Detailed feature augmentation methods within the FAB are shown in Fig 4.

To be more specific:

- Layer swap. In this operation, the column channel of the feature maps is randomly swapped. Since the feature maps extracted by different backbones are similar but not the same, a global noise is introduced by this operation to improve the robustness of the model.
- Channel swap. In this operation, the true bounding boxes mapped to the feature map are randomly swapped. Since the bounding box feature areas contain local receptive fields, they are not restricted to the bounding box areas. The channel swap operation also brings a local noise to improve the robustness of the model.
- Spot cover. The background feature areas are obtained by excluding mapped bounding box feature areas, then we randomly add small black blocks in the background feature areas to produce occlusion. The occlusion information can be transferred to the bounding box areas through the receptive fields of the background feature areas. Therefore, we achieve the Mosaic enhancement for bounding box feature areas without obscuring any valuable target features.

All these feature augmentation operations essentially increase the difference of information obtained by each sub-network, thus improving the diversity of different feature maps.

### Training method

The diversity of backbones as sub-networks is essentially the diversity of parameters. The update of these parameters is closely related to the training methods. Before training, each backbone sub-network is pretrained on public large-scale datasets like ImageNet [24] or MS COCO [25] to acquire basic detection ability and avoid long-time training from scratch. Then, we divide the training of MBDA into four steps to improve the diversity of our sub-networks:

- **Step 1: warm up the parameters of the shared FPN and detectors**. Because these parameters are initialized randomly, we freeze the parameters of backbones to train the shared FPN and detectors on our training dataset.
- **Step 2: train the parameters of the shared FPN**. We freeze the parameters of both the backbones and the detectors to train the shared FPN individually. Considering that the shared FPN is used to improve detection accuracy by feature fusion, i.e., the combination of location and semantic information, we do not adopt any image augmentation or feature augmentation during the training of FPN.
- **Step 3: train the parameters of the backbones and the detectors**. We freeze the parameters of the shared FPN and train the rest of the model. During this stage, we force each detector to find which backbone it belongs to since *N* images are different and have different labels. Each detector needs to make corresponding prediction and we also make this correspondence unchangeable.
- **Step 4: fine-tune the entire network**. We fine-tune the entire network on our training dataset.

Although we adopt multiple backbones in the MBDA, we do not simply sum up their losses to form the final loss function. Considering that different backbones may have different model sizes, convergence speed, etc., we apply the weighted sum of their corresponding losses as:

$$\begin{array}{c} L_{i}\left(x\right)=FL\left({\hat{y}}_{i}^{cls},y_{i}^{cls}\right)+L1\left({\hat{y}}_{i}^{loc},y_{i}^{loc}\right), \end{array}$$ (1)

where *FL*(⋅) is the Focal Loss [52] function for classification, *L*1(⋅) is the smooth L1 loss function for bounding box regression, *x* is the input image, ${\hat{y}}_{i}^{cls},{\hat{y}}_{i}^{loc}$ denote the ground truth label and bounding box location of the augmented *x*, and $y_{i}^{cls},y_{i}^{loc}$ denote the label and bounding box location predicted by the *i*^*th*^ detector respectively. The final loss function can be written as:

$$\begin{array}{c} L\left(x\right)=\sum_{i}w_{i}\cdot L_{i}\left(x\right), \end{array}$$ (2)

where *w*_*i*_ is the weighting factor for the *i*^*th*^ sub-network.

After *t* epochs, we hope to reduce the weight of the sub-network that learns faster on training and validation set, so that the slower trained sub-networks can be trained relatively faster. Therefore, we design the weighting factor to be:

$$\begin{array}{c} w_{i}^{t}=\alpha \tau_{i}^{t}+\beta v_{i}^{t}, \end{array}$$ (3)

where *α* and *β* are hyperparameters (we use *α* = *β* = 1 in the experiment), *τ*_*i*_ and *v*_*i*_ are parameters used to evaluate the convergence of the *i*^*th*^ sub-network on the training set and the validation set, respectively. The convergence parameters can be defined by training/validation loss as:

$$\begin{array}{c} \begin{array}{cc} \tau_{i}^{t} & =min\{1,\frac{L_{i,\tau }^{t}}{\sum_{i}L_{i,\tau }^{t}}exp\left(-\frac{L_{i,\tau }^{t-1}-L_{i,\tau }^{t}}{\sum_{i}\left(L_{i,\tau }^{t-1}-L_{i,\tau }^{t}\right)}\right)\},\\ v_{i}^{t} & =min\{1,\frac{L_{i,v}^{t}}{\sum_{i}L_{i,v}^{t}}exp\left(-\frac{L_{i,v}^{t-1}-L_{i,v}^{t}}{\sum_{i}\left(L_{i,v}^{t-1}-L_{i,v}^{t}\right)}\right)\}, \end{array} \end{array}$$ (4)

where $L_{i,\tau }^{t}$ and $L_{i,v}^{t}$ are the loss of the *i*^*th*^ sub-network calculated on the training and the validation set at the *t*^*th*^ epoch, respectively.

## Experiments

### Experimental setup

In the following sections, we demonstrate various experimental results to illustrate the effectiveness of our proposed method. Below are some experimental setups:

**Data preparation**. The overall dataset is randomly separated according to a ratio of 7:2:1 into three parts: a training set, a validation set, and a test set. Both MBDA and the two baselines are trained on the training set for 5000 epochs. During training, the three models are validated on the validation set for every 50 epochs. Finally, the models with the best validation performance will be tested on the test set.
**Training configuration**. All learnable parameters, including the parameters of all the backbone feature extractors, the shared FPN, and the detectors are jointly tuned by stochastic gradient descent (SGD) for 5000 epochs. The momentum and the weight decaying factor are set to be 0.9 and 5 × 10^−4^, respectively. All the images are resized to 640 × 640 pixels before training and testing. It takes about 147.3 hours to train the proposed model with 400 images (batch size is 32) on one Nvidia RTX 3090 GPU.
**Experimental cases**. We perform two types of detection tasks on the rail defect dataset, one is the 8-category detection task, the other one is the 3-category detection task. In the latter detection task, we regard all defect categories as well as unknown category as one general defect type, i.e., we only care about whether there exist defect in images, not the specific category of the defect.
**Hardware and software implementation**. All the experiments were conducted on Intel(R) Xeon(R) Gold 6226R CPU@2.90GHz and Nvidia RTX 3090 GPU, running an Ubuntu 18.04 operating system. We choose YOLOv5 and Faster R-CNN as our baselines. We do not apply more complex YOLOv5m or YOLOv5l as baselines because we want to achieve real-time detection. All models used are implemented based on PyTorch 1.9.1. YOLOv5 is implemented according to the official GitHub repository, and Faster R-CNN is implemented based on Detectron2 [53].

### Detection result

We test the MBDA with 2 sub-networks (dual-ResNeXt152) and 2 detectors (further studies about sub-network and detector numbers are described in the next section. The detection performance is evaluated by the mean Average Precision with IoU = 0.5 (mAP@.5). The detection result on the rail defect dataset is shown in Table 1. Unless otherwise specified, the mAP@.5 results are averaged from 10 random runs.

**Table 1:** Detection results of different methods over the rail defect datasets.

Method	categories	mAP@.5 (val)	mAP@.5:.95 (val)	mAP@.5 (test)	mAP@.5:.95 (test)	speed (s/img)	fps (img/s)
MBDA (2*ResNeXt152)	3	**0.908**	0.485	0.817	0.382	0.0267	37.45
YOLOv5s	3	0.899	0.462	0.816	0.336	0.0131	76.34
YOLOv5s6	3	0.897	0.466	0.804	0.386	0.0178	56.18
YOLOv5m	3	0.893	0.469	0.806	0.388	0.0237	42.19
FasterRCNN R50	3	0.878	0.464	0.808	**0.387**	0.0690	14.48
FasterRCNN R101	3	0.892	**0.526**	**0.820**	0.373	0.1025	9.76
MBDA (2*ResNeXt152)	8	**0.885**	**0.458**	**0.754**	**0.386**	0.0281	35.59
YOLOv5s	8	0.837	0.321	0.657	0.317	0.0191	52.36
YOLOv5s6	8	0.832	0.348	0.680	0.346	0.0206	48.54
YOLOv5m	8	0.832	0.349	0.697	0.323	0.0241	41.49
FasterRCNN R50	8	0.838	0.345	0.719	0.333	0.1178	8.49
FasterRCNN R101	8	0.840	0.337	0.726	0.322	0.1111	9.00

In the 3-category detection task, our model does not show significant advantages. However, all models’ performance decreased in the 8-category detection task, and our model outperforms the single detection model in both validation and test dataset. The result indicates that the 8-category detection task is harder than detection on more general categories. It is easy to understand because the 3-category detection task only require models to indentify gap, dirt and defect. The 8-category detection detection task, as a comparison, require models to distinguish detailed features of diverse defects with much less samples, thus preventing the model to achieve high detection performance. Our model benefits from the combination of sub-networks as well as image/feature augmentation methods to keep the high detection performance.

**Fig 5:**
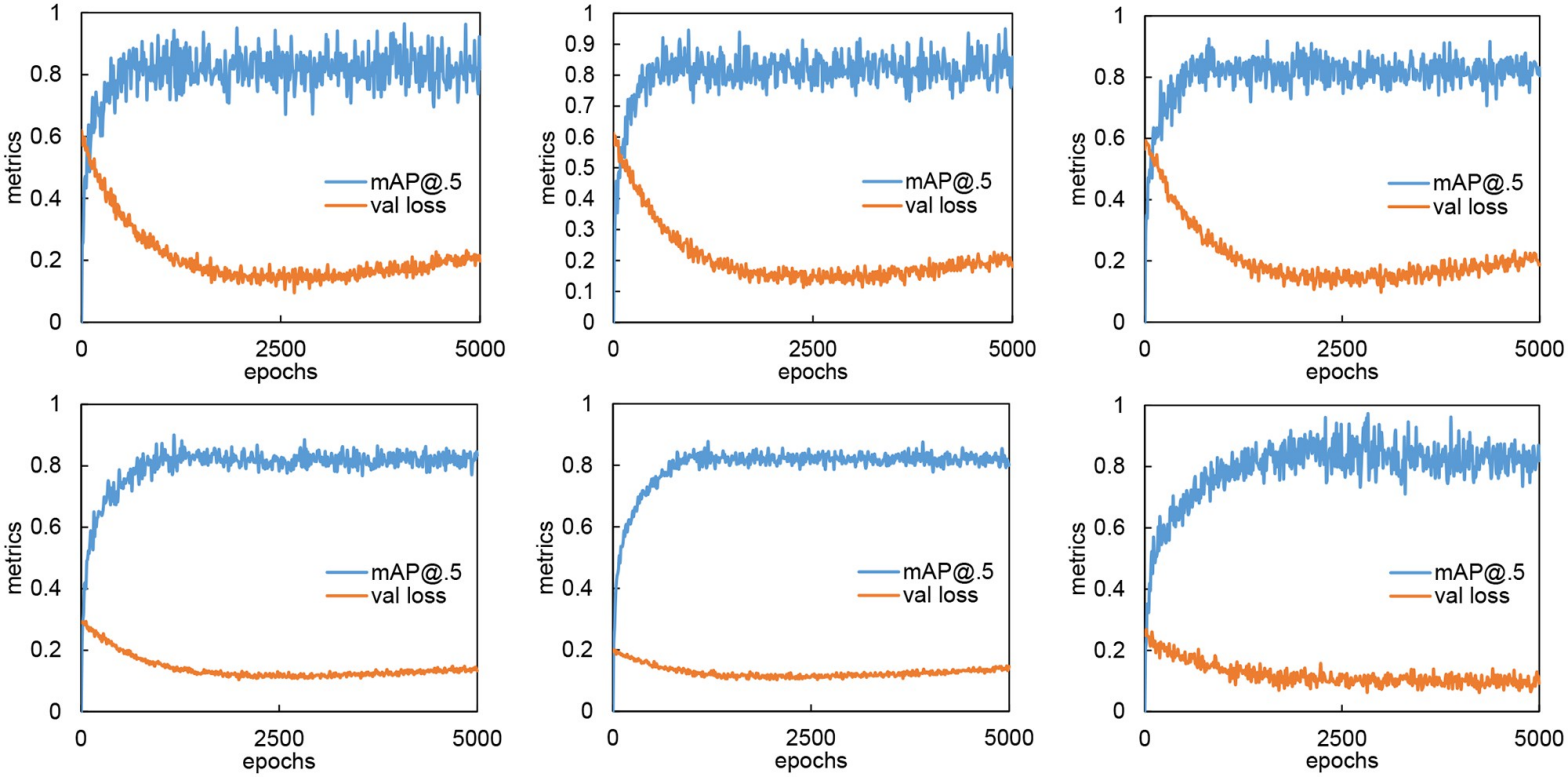
Detection performances and validation losses. The first row from left to right are YOLOv5s, YOLOv5s6, and YOLOv5m. The second row from left to right are FasterRCNN R50, FasterRCNN R101, and MBDA.

The detection performances curves and the validation losses curves are illustrated in Fig 5. We can see from the figure that all models except MBDA have similar validation loss trends that go low at the beginning but start to rise with the growth of epochs. This indicates that all models except MBDA suffer from overfitting.

**Fig 6:**
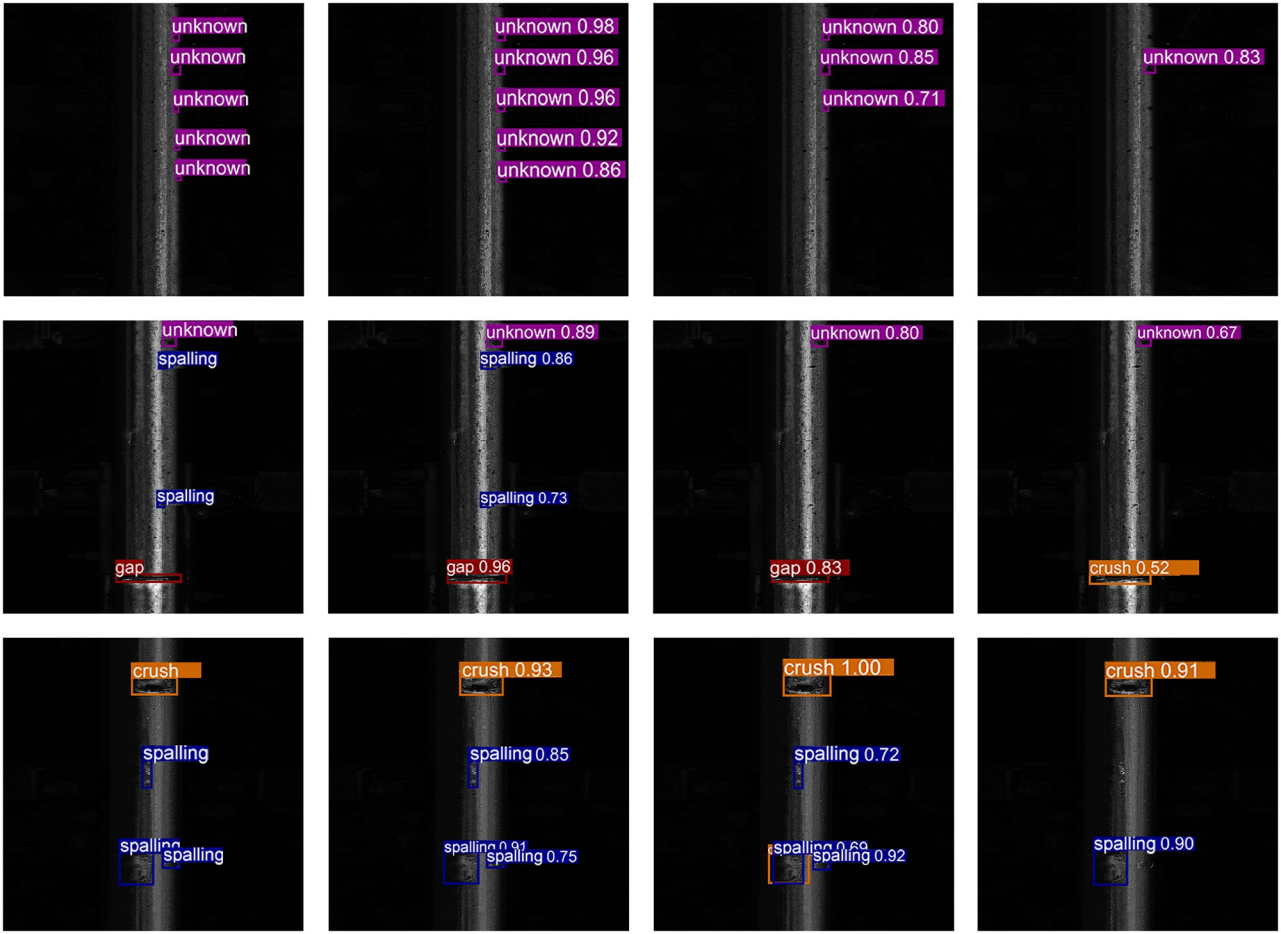
Detection result. From left to right are ground truth, MBDA, FasterRCNN, and YOLO.

The detection results are illustrated in Fig 6. In the figure, MBDA provides detection results closest to ground truth. In the first row, Faster R-CNN neglects two small unknown objects, while YOLO only detects one unknown object. In the second row, Faster R-CNN and YOLO both neglect the two small spalling, and YOLO mistakenly detects the gap as a crush. In the last row, Faster R-CNN detects an extra crush, while YOLO fails to detect the small spalling. The detection result substantiates the fact that our proposed framework performs better detection on rail defects with insufficient training samples.

### MBDA structure analysis

We further study the impact of different types of sub-networks as backbones on 8-category rail defect detection. Subnetworks available in this section are: ResNet50, ResNet101, ResNeXt101, and ResNeXt152. The results are shown in Table 2. In all cases, MBDA performs better than a single network. MBDA with different backbones have similar performance with MBDA with two same backbones (e.g., ResNet50+101 compared to double ResNet 101, and ResNeXt101+152 compared to double ResneXt152), but have fewer parameters (approximately 21% fewer parameters than double Resnet 101, and 13% fewer parameters than double ResneXt152).

**Table 2:** Different MBDA structures.

Backbones	mAP@.5 (val)	mAP@.5 (test)	params.
ResNet50	0.835	0.719	23.5×10^6^
ResNet101	0.840	0.726	42.5×10^6^
ResNeXt101	0.845	0.722	42.0×10^6^
ResNeXt152	0.853	0.731	60.2×10^6^
MBDA (2*ResNet50)	0.839	0.722	48.1×10^6^
MBDA (2*ResNet101)	0.852	0.732	86.1×10^6^
MBDA (ResNet50+101)	0.857	0.739	67.1×10^6^
MBDA (2*ResNeXt101)	0.878	0.754	85.1×10^6^
MBDA (2*ResNeXt152)	**0.885**	**0.754**	121.5×10^6^
MBDA (ResNeXt101+152)	0.883	0.751	103.3×10^6^

**Fig 7:**
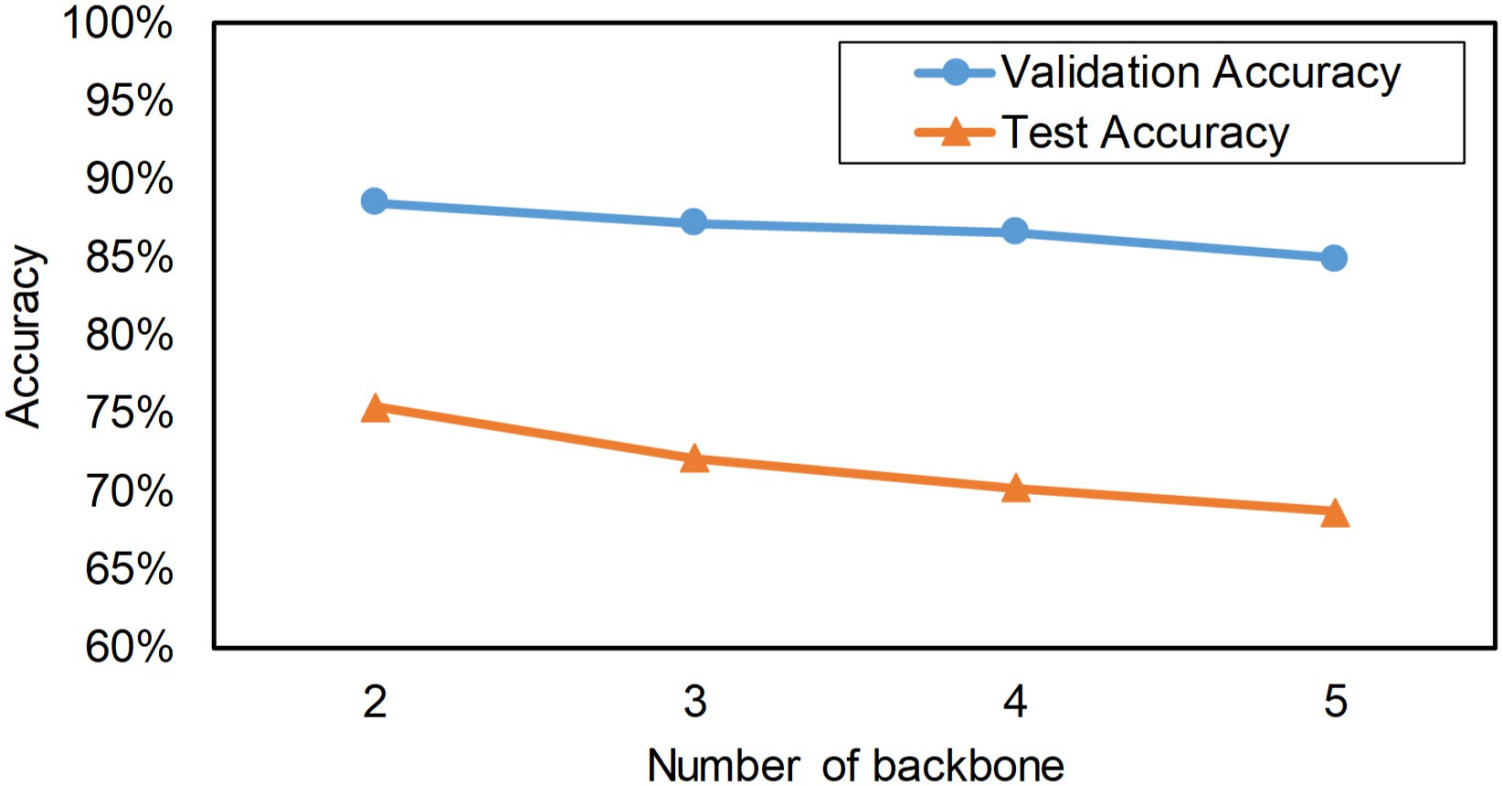
Number of backbones ensembled and their detection precisions.

We also analyze MBDA with 3 or more sub-networks by copying the best performed ResNeXt152 backbone. The analyzed result is illustrated in Fig 7. MBDA’s detection accuracy on the test set gradually decreases with the increasing number of sub-networks. This result has been substantiated by previous research [38]. However, the decrease rate is less than that of previous research, since our sub-networks can partially share features through feature augmentation operation.

### Ablation studies

#### Effectiveness of the IAB

To verify the effectiveness of the image augmentation bag, especially the newly designed object swap and background swap methods, we designed an ablation experiment and the result is shown in Table 3. The result indicates that the newly designed image augmentation operation can achieve enhanced detection performance similar to other image augmentation operations.

**Table 3:** Effectiveness of image augmentation operations. N/A refers to taking no image augmentation method. OS and BS are short for object swap and layer swap, others refer to randomly selected image augmentation methods other than the two newly designed operations.

Backbones	N/A	others	OS	BS	mAP (val)	mAP (test)
2*ResNet101	✓				0.8248	0.703
2*ResNet101		✓			0.8265	0.711
2*ResNet101			✓		0.8256	0.710
2*ResNet101				✓	0.8263	0.714
ResNet50+ResNet101	✓				0.8413	0.712
ResNet50+ResNet101		✓			0.8521	0.716
ResNet50+ResNet101			✓		0.8532	0.714
ResNet50+ResNet101				✓	0.8519	0.718
2*ResNeXt152	✓				0.8612	0.736
2*ResNeXt152		✓			0.8725	0.741
2*ResNeXt152			✓		0.8765	0.742
2*ResNeXt152				✓	0.8762	0.743
ResNeXt101+152	✓				0.8540	0.735
ResNeXt101+152		✓			0.8572	0.740
ResNeXt101+152			✓		0.8589	0.737
ResNeXt101+152				✓	0.8579	0.739

#### Effectiveness of feature augmentation methods

We perform another ablation experiment and the result is shown in Table 4. As is shown in the table, compared to the fixed selection of a certain feature augmentation operation (e.g., solely layer swap, channel swap, or spot cover), models with a random sampling of feature augmentation operations perform better than adopting any fixed feature operation method.

**Table 4:** Effectiveness of feature augmentation operations. LS, CS, SC are short for layer swap, channel swap, spot cover, respectively.

Backbones	LS	CS	SC	FAB	mAP (val)	mAP (test)
2*ResNet101	✓				0.8272	0.705
2*ResNet101		✓			0.8277	0.713
2*ResNet101			✓		0.8256	0.719
2*ResNet101				✓	**0.8398**	**0.724**
ResNet50+ResNet101	✓				0.8487	0.720
ResNet50+ResNet101		✓			0.8514	0.730
ResNet50+ResNet101			✓		0.8501	0.723
ResNet50+ResNet101				✓	**0.8567**	**0.731**
2*ResNeXt152	✓				0.8606	0.737
2*ResNeXt152		✓			0.8756	0.752
2*ResNeXt152			✓		0.8689	0.741
2*ResNeXt152				✓	**0.8794**	**0.753**
ResNeXt101+152	✓				0.8601	0.700
ResNeXt101+152		✓			0.8572	0.738
ResNeXt101+152			✓		0.8568	0.737
ResNeXt101+152				✓	**0.8603**	**0.741**

#### Effectiveness of the combination of IAB and FAB

Table 5 shows the ablation results concerning image augmentation operations and feature augmentation operations. As is shown in the table, solely adopting either image augmentation or feature augmentation could yield better performance on almost all model structures. The best performance could be obtained only when both of the two augmentations are performed.

**Table 5:** The impact of the combination of image augmentation and feature augmentation operations.

Backbones	N/A	IAB	FAB	mAP (val)	mAP (test)
2*ResNet50	✓			0.815	0.689
2*ResNet50		✓		0.825	0.695
2*ResNet50			✓	0.826	0.703
2*ResNet50		✓	✓	**0.839**	**0.722**
2*ResNet101	✓			0.825	0.703
2*ResNet101		✓		0.827	0.714
2*ResNet101			✓	0.840	0.724
2*ResNet101		✓	✓	**0.852**	**0.732**
ResNet50+101	✓			0.841	0.712
ResNet50+101		✓		0.842	0.716
ResNet50+101			✓	**0.857**	0.731
ResNet50+101		✓	✓	**0.857**	**0.739**
2*ResNeXt101	✓			0.832	0.726
2*ResNeXt101		✓		0.856	0.737
2*ResNeXt101			✓	0.860	0.745
2*ResNeXt101		✓	✓	**0.878**	**0.754**
2*ResNeXt152	✓			0.861	0.736
2*ResNeXt152		✓		0.873	0.741
2*ResNeXt152			✓	0.880	0.753
2*ResNeXt152		✓	✓	**0.889**	**0.754**
ResNeXt101+152	✓			0.854	0.735
ResNeXt101+152		✓		0.851	0.737
ResNeXt101+152			✓	0.860	0.741
ResNeXt101+152		✓	✓	**0.883**	**0.751**

## Conclusion

In this paper, we presented the Multi-Backbone Double Augmentation (MBDA) framework to tackle the rail surface defect detection problem. Multiple backbones are ensembled to achieve higher detection performance than a single model. In particular, MBDA with two sub-networks has the best detection performance. In addition, randomly selected image augmentation and feature augmentation operations can increase the diversity of sub-networks, thus improving the robustness of MBDA. The shared FPN as well as the combination of backbones of different parameter levels, on the other hand, helps to reduce the overall parameter and computation cost.

The main limitations of this paper, which are also the limitations of all vision-based defect detection methods, lie in two aspects. First, the proposed method can only detect defects that are recognizable on rail surface. The forumation of rail defects are complex, which makes their manifestations and types vary from each other. This paper covers only a small number of defect types, namely typical surface defects. Second, the detection performance is sensitive to illumination environment. Images too dark or too bright will seriously degrade the detection performance. Possible image proprocessing process may required before training and actually using the proposed method.

## Supporting information

S1 Data All data used to train the model is available at https://github.com/sirlis/RailDefect. (TXT)

S1 File (BST)

## References

[1] Kishore M, Park J, Song S, Kim H, Kwon SG. Characterization of defects on rail surface using eddy current technique. Journal of Mechanical Science and Technology. 2019;33(9):4209–4215. doi: 10.1007/s12206-019-0816-x

[2] Cannon D, Edel KO, Grassie S, Sawley K. Rail defects: an overview. Fatigue & Fracture of Engineering Materials & Structures. 2003;26(10):865–886. doi: 10.1046/j.1460-2695.2003.00693.x

[3] Yang R, Cao S, Kang W, Li J, Jiang X. Mechanism analysis of spalling defect on rail surface under rolling contact conditions. Mathematical Problems in Engineering. 2018;2018.

[4] Liang B, Iwnicki S, Feng G, Ball A, Tran VT, Cattley R. Railway wheel flat and rail surface defect detection by time-frequency analysis. CHEMICAL ENGINEERING. 2013;33.

[5] Toliyat HA, Abbaszadeh K, Rahimian MM, Olson LE. Rail defect diagnosis using wavelet packet decomposition. IEEE Transactions on Industry Applications. 2003;39(5):1454–1461. doi: 10.1109/TIA.2003.816474

[6] Park JW, Lee TG, Back IC, Park SJ, Seo JM, Choi WJ, et al. Rail Surface Defect Detection and Analysis Using Multi-Channel Eddy Current Method Based Algorithm for Defect Evaluation. Journal of Nondestructive Evaluation. 2021;40(3):1–12. doi: 10.1007/s10921-021-00810-9

[7] Jie L, Siwei L, Qingyong L, Hanqing Z, Shengwei R. Real-time rail head surface defect detection: A geometrical approach. In: 2009 IEEE International Symposium on Industrial Electronics. IEEE; 2009. p. 769–774.

[8] Taştimur C, Karaköse M, Akın E, Aydın İ. Rail defect detection with real time image processing technique. In: 2016 IEEE 14th International Conference on Industrial Informatics (INDIN). IEEE; 2016. p. 411–415.

[9] Zhang H, Jin X, Wu QJ, Wang Y, He Z, Yang Y. Automatic visual detection system of railway surface defects with curvature filter and improved Gaussian mixture model. IEEE Transactions on Instrumentation and Measurement. 2018;67(7):1593–1608. doi: 10.1109/TIM.2018.2803830

[10] Yu H, Li Q, Tan Y, Gan J, Wang J, Geng Ya, et al. A coarse-to-fine model for rail surface defect detection. IEEE Transactions on Instrumentation and Measurement. 2018;68(3):656–666. doi: 10.1109/TIM.2018.2853958

[11] Wu Y, Qin Y, Qian Y, Guo F, Wang Z, Jia L. Hybrid deep learning architecture for rail surface segmentation and surface defect detection. Computer-Aided Civil and Infrastructure Engineering. 2022;37(2):227–244. doi: 10.1111/mice.12710

[12] Kaewunruen S, Sresakoolchai J, Zhu G. Machine learning aided rail corrugation monitoring for railway track maintenance. Structural Monitoring and Maintenance. 2021;8(2):151–166.

[13] Sresakoolchai J, Kaewunruen S. Wheel flat detection and severity classification using deep learning techniques. Insight-Non-Destructive Testing and Condition Monitoring. 2021;63(7):393–402. doi: 10.1784/insi.2021.63.7.393

[14] Sresakoolchai J, Kaewunruen S. Prognostics of unsupported railway sleepers and their severity diagnostics using machine learning. Scientific Reports. 2022;12(1):1–10. doi: 10.1038/s41598-022-10062-w 35411031 PMC9001734

[15] Ngamkhanong C, Kaewunruen S. Effects of under sleeper pads on dynamic responses of railway prestressed concrete sleepers subjected to high intensity impact loads. Engineering Structures. 2020;214:110604. doi: 10.1016/j.engstruct.2020.110604

[16] Sresakoolchai J, Kaewunruen S. Detection and severity evaluation of combined rail defects using deep learning. Vibration. 2021;4(2):341–356. doi: 10.3390/vibration4020022

[17] Kaewunruen S, Sresakoolchai J, Stittle H. Machine Learning to Identify Dynamic Properties of Railway Track Components. International Journal of Structural Stability and Dynamics. 2022; p. 2250109. doi: 10.1142/S0219455422501097

[18] Tajbakhsh N, Shin JY, Gurudu SR, Hurst RT, Kendall CB, Gotway MB, et al. Convolutional neural networks for medical image analysis: Full training or fine tuning? IEEE transactions on medical imaging. 2016;35(5):1299–1312. doi: 10.1109/TMI.2016.2535302 26978662

[19] Wei X, Yang Z, Liu Y, Wei D, Jia L, Li Y. Railway track fastener defect detection based on image processing and deep learning techniques: A comparative study. Engineering Applications of Artificial Intelligence. 2019;80:66–81. doi: 10.1016/j.engappai.2019.01.008

[20] Deutschl E, Gasser C, Niel A, Werschonig J. Defect detection on rail surfaces by a vision based system. In: IEEE Intelligent Vehicles Symposium, 2004. IEEE; 2004. p. 507–511.

[21] De Becker D, Dobrzanski J, Justham L, Goh Y. A laser scanner based approach for identifying rail surface squat defects. Proceedings of the Institution of Mechanical Engineers, Part F: Journal of Rail and Rapid Transit. 2021;235(6):763–773. doi: 10.1177/0954409720962252

[22] Lee H, Hong J, Wendimagegn TW, Lee H. Rail corrugation detection and characterization using computer vision. Sensors. 2021;21(24):8335. doi: 10.3390/s21248335 34960429 PMC8709020

[23] Niu M, Song K, Huang L, Wang Q, Yan Y, Meng Q. Unsupervised saliency detection of rail surface defects using stereoscopic images. IEEE Transactions on Industrial Informatics. 2020;17(3):2271–2281.

[24] Deng J, Dong W, Socher R, Li LJ, Li K, Fei-Fei L. Imagenet: A large-scale hierarchical image database. In: 2009 IEEE conference on computer vision and pattern recognition. Ieee; 2009. p. 248–255.

[25] Lin TY, Maire M, Belongie S, Hays J, Perona P, Ramanan D, et al. Microsoft coco: Common objects in context. In: European conference on computer vision. Springer; 2014. p. 740–755.

[26] Zhang Z, Yu S, Yang S, Zhou Y, Zhao B. Rail-5k: a Real-World Dataset for Rail Surface Defects Detection; 2021.

[27] Kang B, Liu Z, Wang X, Yu F, Feng J, Darrell T. Few-shot object detection via feature reweighting. In: Proceedings of the IEEE International Conference on Computer Vision; 2019. p. 8420–8429.

[28] Fan Q, Zhuo W, Tang CK, Tai YW. Few-shot object detection with attention-RPN and multi-relation detector. In: Proceedings of the IEEE Conference on Computer Vision and Pattern Recognition; 2020. p. 4013–4022.

[29] Hu H, Bai S, Li A, Cui J, Wang L. Dense Relation Distillation with Context-aware Aggregation for Few-Shot Object Detection. In: Proceedings of the IEEE Conference on Computer Vision and Pattern Recognition; 2021. p. 10185–10194.

[30] Blockeel H. Hypothesis space. Encyclopedia of Machine Learning. 2011;1:511–513.

[31] Zhang Y, Ren G, Liu X, Gao G, Zhu M. Ensemble learning-based modeling and short-term forecasting algorithm for time series with small sample. Engineering Reports. 2021; p. e12486.

[32] Yu J, Pan R, Zhao Y. High-Dimensional, Small-Sample Product Quality Prediction Method Based on MIC-Stacking Ensemble Learning. Applied Sciences. 2022;12(1):23. doi: 10.3390/app12010023

[33] Krogh PSA, et al. Learning with ensembles: How over-fitting can be useful. In: Proceedings of the 1995 Conference. vol. 8; 1996. p. 190.

[34] Kuncheva LI, Whitaker CJ. Measures of diversity in classifier ensembles and their relationship with the ensemble accuracy. Machine learning. 2003;51(2):181–207. doi: 10.1023/A:1022859003006

[35] Brown G, Wyatt J, Harris R, Yao X. Diversity creation methods: a survey and categorisation. Information fusion. 2005;6(1):5–20. doi: 10.1016/j.inffus.2004.04.004

[36] Adeva JJG, Beresi U, Calvo R. Accuracy and diversity in ensembles of text categorisers. CLEI Electronic Journal. 2005;9(1):1–12.

[37] Sagi O, Rokach L. Ensemble learning: A survey. Wiley Interdisciplinary Reviews: Data Mining and Knowledge Discovery. 2018;8(4):e1249.

[38] Rame A, Sun R, Cord M. MixMo: Mixing Multiple Inputs for Multiple Outputs via Deep Subnetworks. arXiv preprint arXiv:210306132. 2021.

[39] Casado-García Á, Heras J. Ensemble methods for object detection. In: ECAI 2020. IOS Press; 2020. p. 2688–2695.

[40] Körez A, Barışçı N, Çetin A, Ergün U. Weighted ensemble object detection with optimized coefficients for remote sensing images. ISPRS International Journal of Geo-Information. 2020;9(6):370. doi: 10.3390/ijgi9060370

[41] Lin TY, Dollár P, Girshick R, He K, Hariharan B, Belongie S. Feature pyramid networks for object detection. In: Proceedings of the IEEE conference on computer vision and pattern recognition; 2017. p. 2117–2125.

[42] Veit A, Wilber MJ, Belongie S. Residual networks behave like ensembles of relatively shallow networks. Advances in neural information processing systems. 2016;29:550–558.

[43] Attoh-Okine NO. Big data and differential privacy: analysis strategies for railway track engineering. John Wiley & Sons; 2017.

[44] Santur Y, Karaköse M, Akin E. Random forest based diagnosis approach for rail fault inspection in railways. In: 2016 National Conference on Electrical, Electronics and Biomedical Engineering (ELECO). IEEE; 2016. p. 745–750.

[45] He K, Zhang X, Ren S, Sun J. Deep residual learning for image recognition. In: Proceedings of the IEEE conference on computer vision and pattern recognition; 2016. p. 770–778.

[46] Howard AG, Zhu M, Chen B, Kalenichenko D, Wang W, Weyand T, et al. Mobilenets: Efficient convolutional neural networks for mobile vision applications. arXiv preprint arXiv:170404861. 2017.

[47] Solovyev R, Wang W, Gabruseva T. Weighted boxes fusion: Ensembling boxes from different object detection models. Image and Vision Computing. 2021;107:104117. doi: 10.1016/j.imavis.2021.104117

[48] Shorten C, Khoshgoftaar TM. A survey on image data augmentation for deep learning. Journal of Big Data. 2019;6(1):1–48. doi: 10.1186/s40537-019-0197-0 PMC8287113 34306963

[49] Zoph B, Cubuk ED, Ghiasi G, Lin TY, Shlens J, Le QV. Learning data augmentation strategies for object detection. In: European Conference on Computer Vision. Springer; 2020. p. 566–583.

[50] Yun S, Han D, Oh SJ, Chun S, Choe J, Yoo Y. Cutmix: Regularization strategy to train strong classifiers with localizable features. In: Proceedings of the IEEE/CVF International Conference on Computer Vision; 2019. p. 6023–6032.

[51] Breiman L. Bagging predictors. Machine learning. 1996;24(2):123–140. doi: 10.1007/BF00058655

[52] Lin TY, Goyal P, Girshick R, He K, Dollár P. Focal loss for dense object detection. In: Proceedings of the IEEE international conference on computer vision; 2017. p. 2980–2988.

[53] Wu Y, Kirillov A, Massa F, Lo WY, Girshick R. Detectron2; 2019. https://github.com/facebookresearch/detectron2.

