# Correction: Ensemble model for rail surface defects detection

**DOI:** 10.1371/journal.pone.0292773

**Published:** 2023-10-05

**Authors:** Hailang Li, Fan Wang, Junbo Liu, Haoran Song, Zhixiong Hou, Peng Dai

There are errors in the Funding section. The correct Funding statement is: This work was supported by the High-speed Rail Joint Fund Projects under Grant U1934215 and the Scientific Research Projects of China Academy of Railway Sciences under Grant 2020YJ065.
